# Effects of Growth Hormone Replacement Therapy on Bone Mineral Density in Growth Hormone Deficient Adults: A Meta-Analysis

**DOI:** 10.1155/2013/216107

**Published:** 2013-04-17

**Authors:** Peng Xue, Yan Wang, Jie Yang, Yukun Li

**Affiliations:** ^1^Department of Endocrinology, The Third Hospital of Hebei Medical University, 139 Ziqiang Road, Shijiazhuang, Hebei 050000, China; ^2^Department of Epidemiology and Biostatistics, School of Public Health, Hebei Medical University, 361 East Zhongshan Road, Shijiazhuang, Hebei 050000, China

## Abstract

*Objectives*. Growth hormone deficiency patients exhibited reduced bone mineral density compared with healthy controls, but previous researches demonstrated uncertainty about the effect of growth hormone replacement therapy on bone in growth hormone deficient adults. The aim of this study was to determine whether the growth hormone replacement therapy could elevate bone mineral density in growth hormone deficient adults. *Methods*. In this meta-analysis, searches of Medline, Embase, and The Cochrane Library were undertaken to identify studies in humans of the association between growth hormone treatment and bone mineral density in growth hormone deficient adults. Random effects model was used for this meta-analysis. *Results*. A total of 20 studies (including one outlier study) with 936 subjects were included in our research. We detected significant overall association of growth hormone treatment with increased bone mineral density of spine, femoral neck, and total body, but some results of subgroup analyses were not consistent with the overall analyses. *Conclusions*. Our meta-analysis suggested that growth hormone replacement therapy could have beneficial influence on bone mineral density in growth hormone deficient adults, but, in some subject populations, the influence was not evident.

## 1. Introduction

The major role of growth hormone (GH) during childhood is to promote bone growth and linear growth, but GH continues to have important metabolic actions throughout life. Besides growth, GH is known to affect body composition, bone mineralization, and lipid and glucose metabolism [1]. For instance, GH can accelerate bone turnover, which is supported by several lines of evidence. In vitro studies show that GH and its major effector, insulin-like growth factor-1 (IGF-1), are both mitogens for osteoblasts [2, 3].

The condition of GH deficiency (GHD) has been accepted as a definite syndrome, and the clinical and biochemical abnormalities in GHD patients are also well known. They involve mainly the cardiovascular system, lipid metabolism, body composition, mineral metabolism, and quality of life [4, 5]. For example, adult patients with childhood-onset or adult-onset GHD exhibit reduced bone mineral density (BMD) compared with healthy controls [6, 7]. Moreover, clinical studies have shown that the prevalence of fractures is 2.7–3 times higher in GHD patients than in age-matched controls. Data from these studies suggest that the increased risk may be due to GHD rather than other pituitary hormone deficiencies [8, 9].

Many studies have demonstrated that the abnormalities of GHD patients may be reversed by GH replacement therapy, but the evidence is not all conclusive. In particular, the effect of GH treatment on BMD is less clear, though it is well established that GH promotes longitudinal bone growth. Some studies suggest an improvement in BMD [10], some show no effect [11], and others suggest a decrease in BMD related to GH treatment [12]. Moreover, the association between GH treatment and BMD may be influenced by other factors such as gender, treatment time, GH dosage, or geographic location.

We, therefore, undertook a meta-analysis on the effects of GH replacement therapy on BMD based on available studies.

## 2. Methods

### 2.1. Search Strategy and Inclusion Criteria

We systematically searched Medline, Embase, and Cochrane Library for studies written in English (from their commencements to December 2012). The search used the following terms: “growth hormone,” “GH,” “somatotropin,” “bone,” “bone mineral density” and “BMD.” The following three sites of BMD were included in this meta-analysis: spine, femoral neck (FN), and total body (TB).

Studies in humans of the effects of GH treatment on BMD, regardless of sample size, were included if they met the following criteria: (1) data were reported on at least one of the three sites (spine, FN, and TB) of BMD; (2) BMD was measured by dual-energy X-ray absorptiometry (DXA); (3) we only included studies in which mean BMD and standard deviations (SDs), or standard errors (SEs) were available; (4) adult subjects (>16 years old); (5) subjects were patients with GHD who received GH treatment. The excluded studies included reviews, editorials, comments, letters, and abstracts. 

### 2.2. Data Extraction

Two investigators independently reviewed the articles and selected eligible studies according to the inclusion criteria for eligible studies. Irrelevant studies were excluded. For studies with the same population resources or overlapping datasets, the most complete one was included. Study details and data were extracted independently and to a standardized electronic form by two investigators, and discrepancies were adjudicated by a third reviewer until consensus was achieved on every item. The following information was extracted from each study: last name of first author, year of publication, country, subject population, mean, BMD and SDs (or SEs) of subjects at baseline and after GH treatment.

### 2.3. Statistical Analysis

For this meta-analysis, all data should be given as mean and SDs. In those studies, where values of SEs were originally reported, the values of SDs were calculated. When information was reported for more than one subpopulation in one study, each subpopulation was treated as a separate comparison in our meta-analysis. BMDs in the three sites (spine, FN, and TB) were continuous outcomes presented on different scales (g/cm^2^, *z*-scores or *t*-scores), so we used a pooled standardized mean difference (SMD) with 95% confidence intervals (CI) calculated using the final follow-up *P* values to analyze the effects of GH treatment on BMD. All data were initially analyzed with a fixed effects model. If heterogeneity was found, the analysis should be redone using a random effects model. A *P* value of 0.05 was considered statistically significant.

Heterogeneity of the effect across studies was assessed by *Q* statistics, which is distributed as *χ* ^2^ statistics. *I* ^2^ statistics were provided to quantify the percentage of total variation across studies that was attributable to heterogeneity rather than to chance. An *I* ^2^ value >50% represented substantial variability, and heterogeneity was considered to be significant at *P* < 0.10, a conservative standard for meta-analyses. In the presence of heterogeneity, sensitivity analyses were performed to identify the outlier studies. The influence of outliers was also assessed to evaluate the impact of their removal. Moreover, there might be effective modificationcaused by study-level characteristics including treatment time, GH dosage, manufacturer of DXA scanner, and geographic location. Thus, subgroup analyses were further conducted to detect the source of heterogeneity. Treatment time subgroups were defined as ≤2 years or >2 years. GH dosage subgroups were defined as fixed dosage and dosage depending on serum IGF-1 values. Manufacturer of DXA scanner subgroups was defined as GE-Lunar or Hologic Inc, since the studies using other manufacturers of DXA scanner (Norland) are too few. Geographic location subgroups were defined as Europe, America, or Oceania.

We performed a visual inspection of the funnel plot for publication bias. The funnel plot should be asymmetric when there is publication bias and symmetric in the case of no publication bias. We performed Egger and Begg tests to measure the funnel plot asymmetry using a significance level of *P* < 0.05.

All statistical analyses were performed by using STATA 11.0 (Stata Corporation, College Station, TX, USA). The results of our research were reported according to the Preferred Reporting Items for Systematic Reviews and Meta-Analyses (PRISMA) guidelines.

## 3. Results

### 3.1. Studies Included in the Meta-Analysis

Our literature search produced 657 citations written in English, of which we selected 69 for further review of the full text. A total of 49 studies were excluded for unavailable or incomplete data. Finally, 20 unique studies were available for this meta-analysis [10, 11, 13–30]. Of these, 18 studies (included 20 comparisons), 16 studies (included 18 comparisons), and 11 studies (included 12 comparisons) presented data on BMD of spine, FN, and TB, respectively. Tables 1 and 2 summarized the characteristics and the data of the included studies.

In all eligible studies, there were 3 studies separately providing the information on more than one subpopulation. Each subpopulation was treated as a separate comparison. A total of 936 subjects were included in this meta-analysis.

### 3.2. Association between GH Treatment and BMD of Spine

We initially performedthe meta-analysis on all 18 studies (including 20 comparisons) with a fixed effects model. For the presence of significant heterogeneity (*I* ^2^ = 82.9%), the analysis wasredone using a random effects model. The results suggested significant association between GH treatment and increased BMD of spine (SMD = 0.540, 95% CI [0.272, 0.808], *P* < 0.001; *I* ^2^ = 82.9%, *P* < 0.001 for *Q* test).

**Figure 1:**
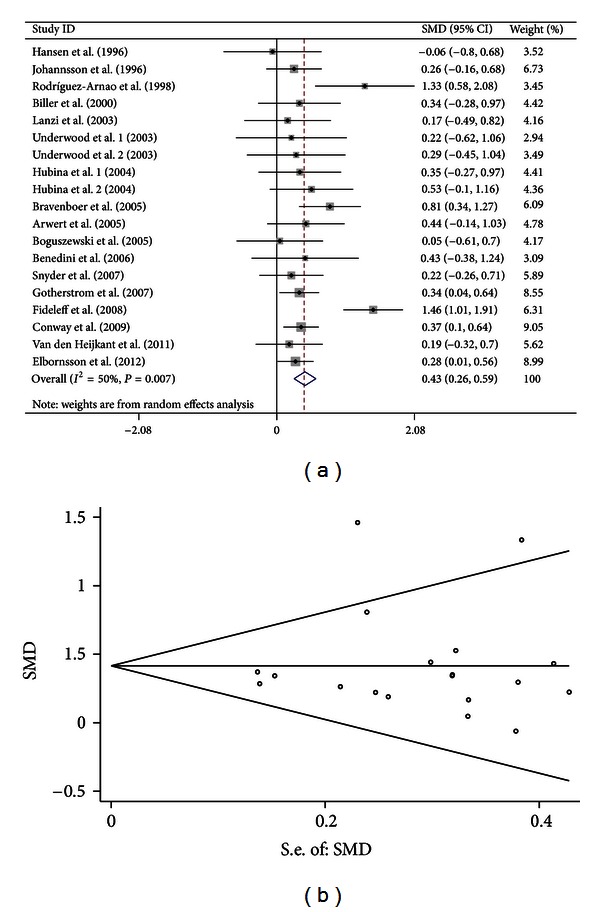
Forest plot and funnel plot for the association between GH treatment and BMD of spine. (a) Forest plot using a random effects model. (b) Funnel plot using Begg methods.

Sensitivity analyses showed that there was an outlier study (study ID: Rota et al.). When the outlier study was omitted, 17 studies (including 19 comparisons) were included in the meta-analysis. The heterogeneity was decreased and the results also suggested significant association between GH treatment and increased BMD of spine (SMD = 0.429, 95% CI [0.263, 0.594],  *P* < 0.001;  *I* ^2^ = 50.0%, *P* = 0.007 for *Q* test) (Figure 1(a)).

To further detect the source of heterogeneity, we performed subgroup analyses stratified by the characteristics (treatment time, GH dosage, manufacturer of DXA scanner, and geographic location) of the subjects. The results did not suggest significant association between GH treatment and BMD of spine in American subjects (SMD = 0.461, 95% CI [−0.049, 0.971], *P* = 0.076; *I* ^2^ = 76.3%, *P* = 0.001 for *Q* test). But a significant association between GH treatment and increased BMD of spine in the other subgroups was found. Significant heterogeneity was removed or decreased in some subgroups.  Table 3 summarizes the subgroup analyses results.

### 3.3. Association between GH Treatment and BMD of FN

Similarly, we performed the meta-analysis on all 16 studies (including 18 comparisons) with arandom effects model. The results suggested significant association between GH treatment and increased BMD of FN (SMD = 0.476, 95% CI [0.190, 0.761], *P* = 0.001; *I* ^2^ = 83.0%, *P* < 0.001 for *Q* test).

**Figure 2:**
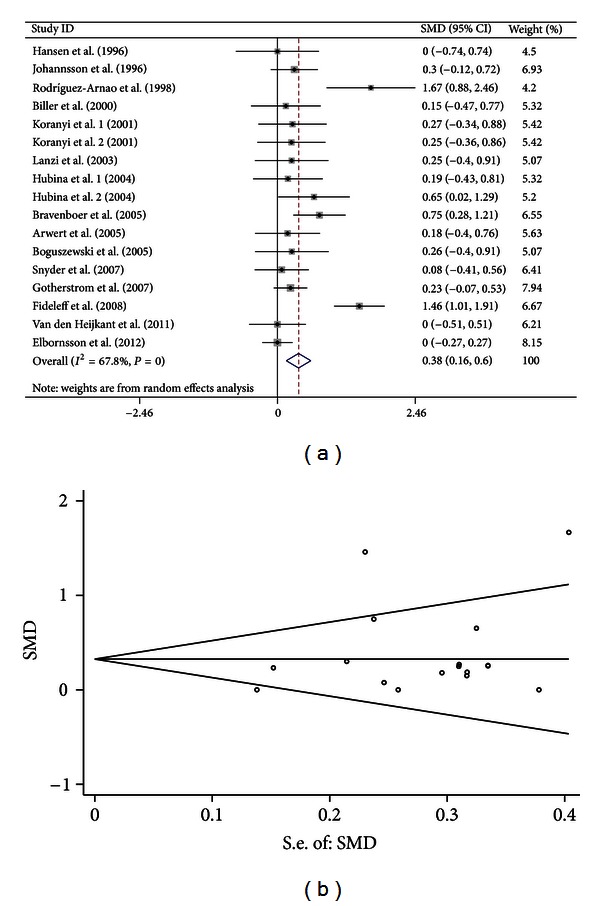
Forest plot and funnel plot for the association between GH treatment and BMD of FN. (a) Forest plot using a random effects model. (b) Funnel plot using Begg methods.

Sensitivity analyses showed that there was also an outlier study (study ID: Rota et al.). When the outlier study was omitted, 15 studies (including 17 comparisons) were included in the meta-analysis. The heterogeneity was decreased and the results also suggested significant association between GH treatment and increased BMD of FN (SMD = 0.377, 95% CI [0.158, 0.595], *P* = 0.001; *I* ^2^ = 67.8%, *P* < 0.001 for *Q* test) (Figure 2(a)).

We also performed subgroup analyses to further detect the source of heterogeneity. The results did not suggest significant association between GH treatment and BMD of FN in subjects treated by GH for ≤2 years (SMD = 0.289, 95% CI [−0.009, 0.587], *P* = 0.057; *I* ^2^ = 51.2%, *P* = 0.045 for *Q* test) and American subjects (SMD = 0.501, 95% CI [−0.227, 1.229], *P* = 0.177; *I* ^2^ = 86.1%, *P* < 0.001 for *Q* test). But significant association between GH treatment and increased BMD of FN in the other subgroups were found. Moreover, the significant heterogeneity was removed or decreased in some subgroups.  Table 4 summarizes the subgroup analyses results.

### 3.4. Association between GH Treatment and BMD of TB

**Figure 3:**
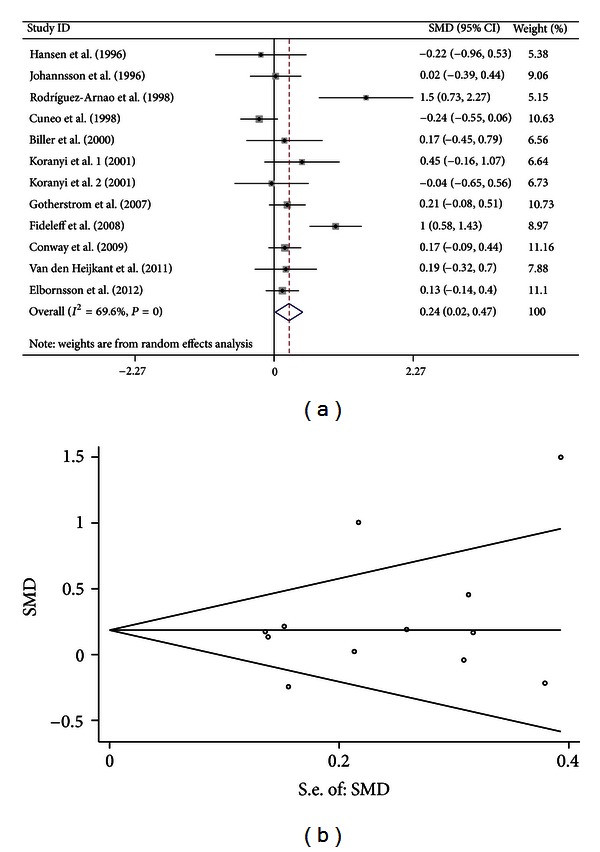
Forest plot and funnel plot for the association between GH treatment and BMD of TB. (a) Forest plot using a random effects model. (b) Funnel plot using Begg methods.

Analogously, we performed the meta-analysis on all 11 studies (including 12 comparisons) with a random effects model. The results suggested significant association between GH treatment and increased BMD of TB (SMD = 0.242, 95% CI [0.019, 0.466], *P* = 0.034; *I* ^2^ = 69.6%, *P* < 0.001 for *Q* test) (Figure 3(a)).

Sensitivity analyses showed that there was no outlier study.

We also performed subgroup analyses to further detect the source of heterogeneity. The results did not suggest significant association between GH treatment and BMD of TB in subjects with treatment time ≤2 years (SMD = 0.159, 95% CI [−0.148, 0.466], *P* = 0.311; *I* ^2^ = 68.1%, *P* = 0.004 for *Q* test), subjects who received fixed GH dosage (SMD = 0.205, 95% CI [−0.406, 0.816], *P* = 0.512; *I* ^2^ = 82.7%, *P* = 0.001 for *Q* test), subjects whose BMD was measured by DXA scanner manufactured by Hologic Inc (SMD = 0.317, 95% CI [−0.101, 0.736], *P* = 0.137; *I* ^2^ = 66.8%, *P* = 0.017 for *Q* test), subjects whose BMD was measured by DXA scanner manufactured by GE-Lunar Inc (SMD = 0.207, 95% CI [−0.083, 0.497], *P* = 0.162; *I* ^2^ = 74.8%, *P* = 0.001 for *Q* test), European subjects (SMD = 0.224, 95% CI [−0.015, 0.463], *P* = 0.066; *I* ^2^ = 51.2%, *P* = 0.045 for *Q* test), American subjects (SMD = 0.618, 95% CI [−0.200, 1.435], *P* = 0.139; *I* ^2^ = 78.9%, *P* < 0.029 for *Q* test,) and Oceanian subjects (SMD = −0.028, 95% CI [−0.438, 0.381], *P* = 0.892; *I* ^2^ = 75.6%, *P* = 0.043 for *Q* test), but as significant association between GH treatment and increased BMD of TB in the other subgroups was found. Moreover, the significant heterogeneity was removed or decreased in some subgroups.  Table 5 summarizes the subgroup analyses results.

### 3.5. Heterogeneity and Publication Bias

Significant heterogeneity was separately observed among the available studies on BMD of spine, FN, and TB. To detect the source of heterogeneity, we performed subgroup analyses stratified by the characteristics of the subjects. Significant heterogeneity was removed or decreased in some subgroups but still existed in other subgroups.

For the 17 studies (with an outlier study excluded) focusing on BMD of spine, both Egger's regression (*P* = 0.789) and Begg methods (*P* = 0.889) did not show publication bias (Figure 1(b)). For the 15 studies (with an outlier study excluded) focusing on BMD of FN, both Egger's regression (*P* = 0.285) and Begg methods (*P* = 0.303) did not show publication bias (Figure 2(b)). For the 11 studies focusing on BMD of TB, both Egger's regression (*P* = 0.309) and Begg methods (*P* = 0.631) did not show publication bias (Figure 3(b)).

## 4. Discussion

In our meta-analysis, we detected an outlier study (study ID: Rota 2008) through sensitivity analyses when we performed the meta-analysis on the association of GH treatment and BMD of spine and FN. In the study mentioned above, patients aged below 30 years and above 50 years were excluded, which might make it an outlier study.

We detected significant overall association between GH treatment and increased BMD of spine, FN, and TB. GH could exert both direct and indirect effects on bone. (1) For direct effects on bone, there was increasing evidence that the GH-IGF axis played a vital role in determining BMD and maintaining bone health and that perturbations in this axis might predispose to the development of osteoporosis. Although GH could act on cells directly through specific receptors [31, 32], most of its anabolic actions were mediated through IGF-1 [33–35]. GH stimulated the secretion of IGF-1, largely from the liver, which then acted in an endocrine fashion. GH also stimulated IGF-1 locally in target tissues such as bone, where it might act in a paracrine or autocrine fashion [36, 37]. Thus, the effect of GH on bone was mediated, at least in part by IGF-1, and bone mass was known to be linked to circulating levels of IGF-1 [38]. In vitro studies had shown that GH-IGF-1 bound to preosteoblasts or mature osteoblasts to induce differentiation and proliferation while also regulating osteoclastic differentiation and activity providing a mechanism to couple bone resorption and formation [39, 40]. In addition, GH also increased biomarkers of bone turnover in normal subjects as well as adults and children with GHD [13, 41]. In almost all of the included studies in our meta-analysis, the serum IGF1 levels of adult GHD patients were significantly increased by the GH treatment, which were listed in Table 2. (2) For indirect effects on bone, It was known that GH had an anabolic effect on skeletal muscle, and it particularly seemed to increase muscle mass and isometric muscle strength when given in physiologically therapeutic doses to GHD patients. Klefter and Feldt-Rasmussen analyzed many trials measuring effects of GH on both muscle and bones [42], and then suggested that there could be a connection between increases in muscle mass and strength and changes in BMD in GHD patients treated with GH. This supported the present physiological concept that the mass and strength of bones were primarily determined by dynamic loads from the skeletal muscles [43, 44].

Significant heterogeneity was found in our meta-analysis. Several study-level variables leading to heterogeneity were defined by subgroup analyses including treatment time, GH dosage, manufacturer of DXA scanner and geographic location. Some results of subgroup analyses were not consistent with the overall analyses.

Firstly, we did not detect significant association between GH treatment and BMD of FN and TB in subjects with treatment time ≤2 yr. GH-IGF1 stimulated bone remodeling which occurred as a biphasic process, dominated initially by bone resorption and only later by bone formation. This biphasic sequence might also explain the initial decrease in BMD reported in several clinical trials [45, 46]. Thus, significant increases in BMD did not usually occur until 12–24 months of treatment, and clinical trials with duration of 24 months or less might not be expected to find significant increases in bone parameters.

Secondly, we did not detect significant association between GH treatment and BMD of TB in subjects received fixed GH dosage (weight- or surface-area-based dosing regimens). Early studies used weight- or surface-area-based dosing regimens that resulted in a higher GH dose than titrating GH dose to normalize the serum IGF-1 level in subsequent years [47]. And the use of dose titration means that it takes longer to establish the patient on a maintenance GH dose. Thus, known differences in the time until response of BMD to GH are recognized. In our meta-analysis, GH treatment time in the studies which determined the GH dosage depending on serum IGF-1 level is mostly longer than that isthe studies which used fixed GH dosage. So, the effect of the former dosing regimens on BMD might be more evident.

Thirdly, we did not detect significant association between GH treatment and BMD of TB in the subjects whose BMD was measured by DXA scanner manufactured by Hologic Inc or GE-lunar but got a significant overall association between GH treatment and increased BMD of TB. Absolute values of BMD, using DXA, might differ between instruments from different manufacturers. previous study performed a comparison of longitudinal measurements in the spine and proximal femur using lunar and Hologic instruments [48]. Despite the significant correlations, the agreement between the two densitometers was not high and there might be significant errors in individual subjects if one uses measurements from one densitometer to predict the change in BMD using the scanner of the other manufacturer. Furthermore, there were three studies (study ID: Arwert 2005, Gotherstrom 2007, Elbornsson 2012) which lasted for more than 10 years included in our meta-analysis. In the three studies, the operating criterion of BMD measurements changed partly during the GH treatment. In the study reported by Arwert et al., BMD measurements were performed with Norland XR-26 at the beginning of the study, and with Hologic QDR-4500 at the end of the study, but they tried to resolve this problem. They measured the European Spine Phantom (ESP) on both devices, and the results of ESP measurements showed very similar BMD values. In the study reported by Gotherstrom et al., the software versions of Lunar DPX-L were changed several times (from 1.1 to finally 1.35) during the study, but the version 1.33 was generally used during the large period of the study. In the study reported by Elbornsson et al., BMD measurements were performed with LUNAR DPX-L scanner at the beginning of the study and with LUNAR Prodigy scanner at the end of the study. They measured 31 subjects' BMD with both scanners on the same day, and the BMD values of the subjects were not significantly different between scanners.

Fourthly, we did not detect significant association between GH treatment and BMD of spine, F, and TB in American subjects. In addition, we did not detect significant association between GH treatment and BMD of TB in European and Oceanian subjects but got a significant overall association between GH treatment and increased BMD of TB. Although GH was a major regulator of IGF-1 concentrations, other factors such as nutrition and insulin concentration were also important in its regulation. In different countries or geographic location, the nutrition or insulin concentration of GHD patients might also be different.

Heterogeneity was removed or decreased in some subgroups but still existed in other subgroups. Thus, in addition to treatment time, GH dosage, manufacturer of DXA scanner and geographic location, there might be other factors leading to heterogeneity. For instance, previous study indicated that gender might influence the association between GH treatment and BMD, and several studies suggested that men had a greater treatment response to GH replacement than women. The mechanisms underlying these gender differences were not fully understood, but sex hormones might play a role [49, 50]. However, there were too few studies analyzing the results of GH treatment separately for males and females in the studies included in our meta-analysis, which made it impossible to undertake subgroup analyses stratified by gender. For the studies focus on BMD of spine, FN and TB, both Egger's regression and Begg methods did not show publication bias.

In our study, we mainly investigated the effects of GH treatment on BMD in adult GHD patients. Besides its beneficial effects on bone, GH treatment is also suggested to alleviate at least some of the aspects of the reduced physical and psycHological health associated with GHD in adult life [51, 52]. Severe quality of life (QoL) impairment is evident in a significant proportion of adults with GHD, and the beneficial effects of physiological GH replacement on QoL in affected individuals are well documented [53–55]. However, the risk of adverse effects may increase in the GHD adults treated with GH, such as oedema, joint stiffness, and carpal tunnel syndrome [54]. Moreover, serum IGF1 levels increase in the GHD patients with GH treatment. In the general population, higher circulating IGF1 levels are associated with increased incidence of prostate, colorectal, and premenopausal breast cancer [56]. To date, however, there have been no published long-term studies in adults with GHD treated with GH with respect to the development of nonpituitary malignancies.

The present study has some limitations that should be considered. Firstly, because only studies that were indexed by the selected databases were included for data analysis, some relevant published studies or unpublished studies might be missed, which might have biased our results. Secondly, our meta-analysis only included adult subjects since it was inappropriate to pool data from studies in children, where growth had a major effect of GH therapy; with studies in adults, this was not the case.

## 5. Conclusions

Considered together, these studies seem to indicate that GH treatment had beneficial influence on BMD in GHD adults, but in some subject populations, the influence was not evident.

## Figures and Tables

**Table 1:** Patient characteristics in included studies.

References	Countries	Study subjects	Criteria of GHD	Treatment time	GH usage
Hansen et al., 1996 [13]	Denmark	9 males and 5 females aged 31–57	<10 mU/L in ITT^a^	1 yr	2.0 IU/m^2^·day

Johannsson et al., 1996 [14]	Sweden	24 males and 20 females aged 23–66	<5 mU/L in ITT	2 yr	First 4 weeks: 0.1 IU/kg·week Thereafter: 0.25 IU/kg·week

Rodríguez-Arnao et al., 1998 [15]	UK	18 males and 17 females aged 21.1–59.9	<10 mU/L in ITT	1 yr	First 4 weeks: 0.125 IU/kg·week Thereafter: 0.25 IU/kg·week

Cuneo et al., 1998 [16]	Australia	50 males and 33 females aged 41.2 ± 1.5	<5 mU/L in ITT	1 yr	First month: 0.125 IU/kg·week Thereafter: 0.25 IU/kg·week

Biller et al., 2000 [17]	USA	38 males aged 48.9 ± 2.0	NA^b^	1.5 yr	Initial dose: 10 *μ*g/kg·day Then adjusted accordingly to the serum IGF-I values

Koranyi et al., 2001 [18]	Sweden	28 males and 14 females aged 17–61	NA	5 yr	Initial dose: 0.25 IU/kg·week Then adjusted accordingly to the serum IGF-I values

Lanzi et al., 2003 [19]	Italy	10 males and 8 females aged 17–50	<9 *μ*g/L in ARG^c^	0.5 yr	Initial dose: 4 *μ*g/kg·day Then adjusted accordingly to the serum IGF-I values

Underwood et al., 2003 [20]	USA	39 males and 25 females aged 23.8 ± 4.2	<5 *μ*g/L in clonidine and L-dopa stimulation tests	2 yr	25 *μ*g/kg·day or 12.5 *μ*g/kg·day

Hubina et al., 2004 [21]	Hungary	11 males and 9 females aged 22–67	<3 *μ*g/L in ITT or ARG	3 yr	1.2 IU/day (average dose)

Bravenboer et al., 2005 [22]	The Netherlands	38 males aged 20–35	<7 *μ*g/L in ITT or GHRH^d^	5 yr	0.67 mg/m^2^·day

Arwert et al., 2005 [23]	The Netherlands	23 males aged 20–40	<7 *μ*g/L in ITT or GHRH	10 yr	First 0.5 yr: 1.2 or 3 IU/m^2^·day 0.5–2 yr: 2 IU/m^2^·day Then adjusted accordingly to the serum IGF-I values

Boguszewski et al., 2005 [24]	Brazil	7 males and 11 females aged 21–58	<3 *μ*g/L in ITT	1 yr	0.6 IU/day

Benedini et al., 2006 [25]	Italy	6 males and 6 females aged 29–54	<9 *μ*g/L in ARG and GHRH	1 yr	Initial dose: 0.25 mg/day (for men) or 0.4 mg/day (for women) Then adjusted accordingly to the serum IGF-I values

Snyder et al., 2007 [26]	USA	20 males and 13 females aged 29–54	<2.4 *μ*g/L in ITT or ARG	2 yr	Initial dose: 2 *μ*g/kg·day Then adjusted accordingly to the serum IGF-I values

Gotherstrom et al., 2007 [27]	Sweden	52 males and 35 females aged 22–74	<3 *μ*g/L in ITT	10 yr	64 patients: the initial dose was 0.25 IU/kg·week and then individualized 23 patients: the dose was individualized from the start of the treatment

Rota et al., 2008 [28]	Italy	35 males and 29 females aged 30–50	<9 *μ*g/L in ARG and GHRH	2 yr	Initial dose: 4 *μ*g/kg·day Then adjusted accordingly to the serum IGF-I values

Fideleff et al., 2008 [10]	Argentina	22 males and 26 females aged 18–66	<0.14 pmol/L in ITT	4 yr	Initial dose: 0.1 mg/day Then adjusted accordingly to the serum IGF-I values

Conway et al., 2009 [29]	Australia	65 males and 44 females aged 21.1 ± 2.3	<3 *μ*g/L in ITT	2 yr	Initial dose: 0.2 mg/day (for men) or 0.4 mg/day (for women) Then adjusted accordingly to the serum IGF-I values

van den Heijkant et al., 2011 [11]	The Netherlands	12 males and 8 females aged 23.9 ± 3.0	<3 *μ*g/L in ITT	2 yr	Initial dose: 0.1 mg/m^2^·day Then adjusted accordingly to the serum IGF-I values

Elbornsson et al., 2012 [30]	Sweden	72 males and 54 females aged 22–74	<3 *μ*g/L in ITT or GHRH	15 yr	64 patients: the initial dose was 0.25 IU/kg·week and then individualized 62 patients: the dose was individualized from the start of the treatment

^a^Insulin tolerance test; ^b^not available; ^c^arginine test; ^d^GH-releasing hormone.

**Table 2:** BMD measurements and outcomes.

References	DXA scanner manufacturer	BMD sites	CV^a ^of BMD measurements	Baseline BMD (mean ± SD)	Posttreatment BMD (mean ± SD)	BMD scales	Serum IGF1 changes
Hansen et al., 1996 [13]	Hologic Inc.	Spine FN TB	0.6% 1.4% 1.6%	0.960 ± 0.170 0.920 ± 0.150 1.100 ± 0.070	0.950 ± 0.150 0.920 ± 0.150 1.080 ± 0.110	g/cm^2^	Increased 263 ± 98%

Johannsson et al., 1996 [14]	GE-Lunar	Spine FN TB	0.5% 1.6% 0.4%	1.170 ± 0.186 0.944 ± 0.133 1.164 ± 0.133	1.218 ± 0.179 0.986 ± 0.146 1.167 ± 0.119	g/cm^2^	Increased from 77 ± 7 to 302 ± 15 *μ*g/L

Rodríguez-Arnao et al., 1998 [15]	Hologic Inc.	Spine FN TB	1.0% 1.8% NA^b^	1.000 ± 0.030 0.790 ± 0.030 1.150 ± 0.020	1.040 ± 0.030 0.840 ± 0.030 1.180 ± 0.020	g/cm^2^	Increased significantly with 31% of the total patients showing IGF1 levels above the age-corrected limit of normal

Cuneo et al., 1998 [16]	GE-Lunar	TB	<3.8%	1.149 ± 0.155	1.120 ± 0.092	g/cm^2^	Increased from 100 to 280 ± 20 *μ*g/L

Biller et al., 2000 [17]	Hologic Inc.	Spine FN TB	NA NA NA	0.700 ± 0.134 0.920 ± 0.165 1.189 ± 0.134	0.751 ± 0.161 0.948 ± 0.206 1.219 ± 0.215	g/cm^2^	NA

Koranyi et al., 2001 (1) [18]	GE-Lunar	FN TB	NA NA	0.919 ± 0.206 1.111 ± 0.110	0.975 ± 0.211 1.161 ± 0.110	g/cm^2^	Increased from 85.8 ± 15.9 to 295.1 ± 36.3 *μ*g/L

Koranyi et al., 2001 (2) [18]	GE-Lunar	FN TB	NA NA	1.012 ± 0.142 1.214 ± 0.101	1.048 ± 0.147 1.210 ± 0.092	g/cm^2^	Increased from 135.4 ± 15.5 to 283.3 ± 28.9 *μ*g/L

Lanzi et al., 2003 [19]	Hologic Inc.	Spine FN	0.5% NA	−1.080 ± 0.180 −0.900 ± 0.370	−1.050 ± 0.180 −0.810 ± 0.340	*t*-score	Increased from 102.94 ± 16.93 to 226.17 ± 17.10 *μ*g/L

Underwood et al., 2003 (1) [20]	GE-Lunar	Spine	NA	−1.340 ± 1.360	−1.050 ± 0.180	*z*-score	IGF1 SDS increased from −5.2 ± 2.6 to −0.6 ± 1.5

Underwood et al., 2003 (2) [20]	GE-Lunar	Spine	NA	−1.010 ± 1.410	−0.610 ± 1.300	*z*-score	IGF1 SDS increased from −3.8 ± 1.5 to 1.2 ± 1.5

Hubina et al., 2004 (1) [21]	Hologic Inc.	Spine FN	0.35% NA	−1.690 ± 1.480 −0.750 ± 2.147	−1.210 ± 1.252 −0.380 ± 1.789	*t*-score	IGF1 SDS increased from −2.53 ± 0.85 to 0.12 ± 0.41

Hubina et al., 2004 (2) [21]	Hologic Inc.	Spine FN	0.35% NA	−1.200 ± 2.147 −0.450 ± 0.894	−0.210 ± 1.565 0.210 ± 1.118	*t*-score	IGF1 SDS increased from −3.61 ± 0.96 to 0.74 ± 0.28

Bravenboer et al., 2005 [22]	Norland	Spine FN	2.4% 2.3%	0.920 ± 0.140 0.820 ± 0.150	1.050 ± 0.180 0.940 ± 0.170	g/cm^2^	Increased from 9.9 ± 5.7 to 27.2 ± 11.8 nM

Arwert et al., 2005 [23]	Hologic Inc.	Spine FN	NA 2.1%	0.900 ± 0.150 0.780 ± 0.120	0.960 ± 0.120 0.800 ± 0.100	g/cm^2^	Increased from 9.7 ± 2.1 to 26.6 ± 6.1 nM

Boguszewski et al., 2005 [24]	GE-Lunar	Spine FN	1.2% 1.5%	1.121 ± 0.210 0.903 ± 0.170	1.131 ± 0.210 0.948 ± 0.180	g/cm^2^	Increased from 76.9 ± 70.4 to 133.7 ± 134.1 *μ*g/L

Benedini et al., 2006 [25]	Hologic Inc.	Spine	NA	0.950 ± 0.130	1.000 ± 0.100	g/cm^2^	Increased from 60 ± 29 to 151 ± 49 *μ*g/L

Snyder et al., 2007 [26]	Hologic Inc.	Spine FN	0.37–0.51%	1.050 ± 0.130 0.820 ± 0.130	1.080 ± 0.140 0.830 ± 0.130	g/cm^2^	IGF1 SDS increased from −1.65 ± 0.92 to 0.20 ± 1.40

Gotherstrom et al., 2007 [27]	GE-Lunar	Spine FN TB	0.5% 0.6% 0.4%	1.161 ± 0.205 0.939 ± 0.159 1.163 ± 0.140	1.243 ± 0.270 0.976 ± 0.159 1.194 ± 0.149	g/cm^2^	Increased from 99.5 ± 6.6 to 223.3 ± 9.8 *μ*g/L

Rota et al., 2008 [28]	Hologic Inc.	Spine FN	1.0% 1.5%	−1.700 ± 0.200 −0.700 ± 0.200	−1.300 ± 0.100 −0.400 ± 0.100	*z*-score	Increased 174.1 ± 31.2% in men and 301.7 ± 97.1% in women

Fideleff et al., 2008 [10]	GE-Lunar	Spine FN TB	NA NA NA	−1.300 ± 1.386 −1.200 ± 1.386 −1.000 ± 1.386	0.300 ± 0.693 0.400 ± 0.693 0.100 ± 0.693	*z*-score	IGF1 SDS increased from −4.54 ± 0.42 to 0.36 ± 0.25

Conway et al., 2009 [29]	Hologic Inc.	Spine TB	NA NA	0.910 ± 0.130 0.980 ± 0.110	0.960 ± 0.140 1.000 ± 0.120	g/cm^2^	Increased from 132.9 ± 128.1 to 361.6 ± 259.5 *μ*g/L

Van den Heijkant et al., 2011 [11]	Hologic Inc.	Spine FN TB	NA NA NA	0.960 ± 0.110 0.840 ± 0.130 1.010 ± 0.100	0.980 ± 0.100 0.840 ± 0.130 1.030 ± 0.110	g/cm^2^	NA

Elbornsson et al., 2012 [30]	GE-Lunar	Spine FN TB	<1.5%	1.170 ± 0.224 0.940 ± 0.112 1.170 ± 0.112	1.230 ± 0.190 0.940 ± 0.190 1.190 ± 0.190	g/cm^2^	Increased from 103 ± 6 to 183 ± 7 *μ*g/L

^a^Coefficient of variation; ^b^not available.

**Table 3:** Subgroup analyses results of the association between GH treatment and BMD of spine

Subgroups	Effects of GH treatment on BMD			Heterogeneity		
	SMD	95% CI	*P *	*I* ^2^ (%)	χ^2^	*P *
Treatment time						
≤2 yr	0.311	0.159–0.463	0.000	0.0	9.62	0.565
>2 yr	0.597	0.275–0.919	0.000	73.6	22.69	0.001
GH dosage						
Fixed dosage	0.429	0.172–0.686	0.001	34.2	12.16	0.144
Dosage depending on serum IGF-1 values	0.429	0.203–0.655	0.000	62.2	23.79	0.005
Manufacturer of DXA scanner						
Hologic Inc	0.362	0.204–0.520	0.000	0.0	9.15	0.518
GE-lunar	0.440	0.097–0.783	0.012	74.7	23.72	0.001
Geographic location^a^						
Europe	0.385	0.232–0.537	0.000	13.9	12.78	0.308
America	0.461	−0.049–0.971	0.076	76.3	21.07	0.001

^a^There was only one study including Oceanian subjects.

**Table 4:** Subgroup analyses results of the association between GH treatment and BMD of FN.

Subgroups	Effects of GH treatment on BMD			Heterogeneity		
	SMD	95% CI	*P *	*I* ^2^ (%)	χ^2^	*P *
Treatment time						
≤2 yr	0.289	−0.009–0.587	0.057	51.2	14.36	0.045
>2 yr	0.440	0.119–0.761	0.007	76.9	34.70	0.000
GH usage						
Fixed dosage	0.520	0.178–0.861	0.003	56.5	13.79	0.032
Dosage depending on serum IGF-1 values	0.289	0.007–0.571	0.045	72.3	32.51	0.000
Manufacturer of DXA scanner						
Hologic Inc	0.306	0.018–0.595	0.037	49.7	15.89	0.044
GE-lunar	0.392	0.026–0.758	0.036	80.2	30.34	0.000
Geographic location^a^						
Europe	0.313	0.117–0.508	0.002	48.0	23.08	0.027
America	0.501	−0.227–1.229	0.177	86.1	21.54	0.000

^a^There was no study including Oceanian subjects.

**Table 5:** Subgroup analyses results of the association between GH treatment and BMD of TB.

Subgroups	Effects of GH treatment on BMD			Heterogeneity		
	SMD	95% CI	*P *	*I* ^2^ (%)	χ^2^	*P *
Treatment time						
≤2 yr	0.159	−0.148–0.466	0.311	68.1	18.81	0.004
>2 yr	0.352	0.015–0.688	0.041	70.9	13.76	0.008
GH usage						
Fixed dosage	0.205	−0.406–0.816	0.512	82.7	17.35	0.001
Dosage depending on serum IGF-1 values	0.283	0.076–0.491	0.007	52.0	14.59	0.042
Manufacturer of DXA scanner						
Hologic Inc	0.317	−0.101–0.736	0.137	66.8	12.06	0.017
GE-lunar	0.207	−0.083–0.497	0.162	74.8	23.77	0.001
Geographic location						
Europe	0.224	−0.015–0.463	0.066	51.2	14.34	0.045
America	0.618	−0.200–1.435	0.139	78.9	4.75	0.029
Oceania	−0.028	−0.438–0.381	0.892	75.6	4.10	0.043
